# Population Genetics of Two Asexually and Sexually Reproducing Psocids Species Inferred by the Analysis of Mitochondrial and Nuclear DNA Sequences

**DOI:** 10.1371/journal.pone.0033883

**Published:** 2012-03-27

**Authors:** Dan-Dan Wei, Ming-Long Yuan, Bao-Jun Wang, An-Wei Zhou, Wei Dou, Jin-Jun Wang

**Affiliations:** Key Laboratory of Entomology and Pest Control Engineering, College of Plant Protection, Southwest University, Chongqing, People's Republic of China; Brigham Young University, United States of America

## Abstract

**Background:**

The psocids *Liposcelis bostrychophila* and *L. entomophila* (Psocoptera: Liposcelididae) are found throughout the world and are often associated with humans, food stores and habitations. These insects have developed high levels of resistance to various insecticides in grain storage systems. However, the population genetic structure and gene flow of psocids has not been well categorized, which is helpful to plan appropriate strategies for the control of these pests.

**Methodology/Principal Findings:**

The two species were sampled from 15 localities in China and analyzed for polymorphisms at the mitochondrial DNA (*Cytb*) and ITS (ITS1-5.8S-ITS2) regions. In total, 177 individual *L. bostrychophila* and 272 individual *L. entomophila* were analysed. Both *Cytb* and ITS sequences showed high genetic diversity for the two species with haplotype diversities ranged from 0.154±0.126 to 1.000±0.045, and significant population differentiation (mean *F*_ST_ = 0.358 for *L. bostrychophila*; mean *F*_ST_ = 0.336 for *L. entomophila*) was also detected among populations investigated. A Mantel test indicated that for both species there was no evidence for isolation-by-distance (IBD). The neutrality test and mismatch distribution statistics revealed that the two species might have undergone population expansions in the past.

**Conclusion:**

Both *L. bostrychophila* and *L. entomophila* displayed high genetic diversity and widespread population genetic differentiation within and between populations. The significant population differentiation detected for both psocids may be mainly due to other factors, such as genetic drift, inbreeding or control practices, and less by geographic distance since an IBD effect was not found.

## Introduction

Genetic variability is considered to be the foundation of evolution, and it can be affected by a variety of factors, such as mutation rates, effective population size, and gene flow [1]. The current distribution of genetic variation within a species is caused by its demographic history and a combination of the factors previously mentioned. Gene flow is constraint on local genetic differentiation and the adaptation between populations, and low gene flow between populations can lead to genetic subdivision of populations [2]. Decreased genetic diversity in a population can cause a reduction in individual fitness through the expression of inbreeding depression-like effects and further reduce the effective population size (*N*e) [3]. Levels of genetic diversity in natural populations primarily reflect long-term processes in which a balance is achieved between the generation of diversity by new mutations and loss of diversity by drift. Food resources, dispersal ability (active or passive), geographic isolation, exposure to pesticides and other environmental factors can influence and shape a pest's population structure [4]. A thorough understanding of genetic diversity and population structure on both large and local geographic scales are crucial. Different evolutionary forces and environmental factors can provide us with important information about population dynamics and help to develop effective pest management strategies.

Mitochondrial DNA (mtDNA), due to its accelerated rate of evolution, simple maternally inherited, and lacking of recombination, has been used as a powerful and efficient way to study genetic diversity in insect populations at both the inter- and intra-specific levels, especially for cytochrome b (*Cytb*) gene [5], [6]. The ribosomal internal transcribed spacer (ITS) region, have been widely used for population genetic studies [7], [8] and species identification [9], [10] due to its highly conserved flanking regions (and so ease of use in PCR) and rapid evolution rates. However, pseudogenic ITS regions must be critically considered in population level and phylogenetic studies as their utility is controversial and sometimes they may cause erroneous analyses [11]. Fortunately, the presence of conserved 5.8 S motifs and secondary structure can provide a useful and swift tool for the pseudogenic ITS regions identification, thus easily to exclude these pseudogenes from analyses [12].

The small, wingless psocids, *Liposcelis bostrychophila* Badonnel and *L. entomophila* (Enderlein) are found throughout the world and are often associated with humans, food stores and habitations [13]. There is a growing awareness of the important pest status of psocids due to the economic losses suffered by psocid infestation of stored grains, as well as from their contamination of food and commodities [13]–[15]. Recently, they have become a new risk for global food security and safety [16]. *L. bostrychophila* and *L. entomophila* are similar in morphology and often commix within the same ecosystems. As the closest free living relatives of parasitic lice, these two species can also transmit harmful microorganisms, including fungi and bacteria [17], [18]. Compared to *L. bostrychophila*, *L. entomophila* has been found to be more commonly and more widely distributed in grain storage systems in China. Unlike the sexual reproduction undertaken by *L. entomophila*, *L. bostrychophila* is an obligatory parthenogenetic species and displays considerable adaptability to deal with local or temporary situations [19]. In *L. bostrychophila*, parthenogenesis can maintain heterozygosity but with the loss of variation [20]. Asexual reproduction is commonly thought to be associated with low genetic diversity in animals (due to lacking of recombination) and such organisms have generally been regarded as evolutionary dead ends [21], [22]. However, the apparent lower genetic diversity of asexual animals compared to closely relate sexual species has been called into question [23], [24]. Paradoxically, against the background of its parthenogenetic characteristics, *L. bostrychophila* also shows a considerable degree of morphological and physiological variation both between and within clones [19], [25].

On the other hand, numerous studies have been conducted on psocid physiology, biochemistry, and molecular biology in China and worldwide [26]–[29]; however, the population genetic structure of psocids has not been well established. Population genetic variation for *L. bostrychophila* has been investigated using allozymes [19], and randomly amplified polymorphic DNA (RAPDs) [30]. Microsatellite markers have been characterised for *L. decolor* and cross-amplified in five other *Liposcelis* species of economic importance [31] and these same microsatellites have been used to investigate the genetic structure and gene flow of *L. decolor* in Australia [32]. To our knowledge, no information is available on the population genetic structure of *L. entomophila*. As little is known of the population genetics of *L. bostrychophila* or *L. entomophila* in China, we undertook a genetic study to investigate the genetic diversity, the population genetic structure and population demographic history of the two psocids using mitochondrial *Cytb* and ITS sequences.

## Materials and Methods

### Ethics Statement

No specific permits were required for the described field studies, and no specific permissions were required for these locations/activities. We confirm that these locations are not privately-owned or protected in any way and the field studies did not involve endangered or protected species.

### Sampling and DNA extraction

**Figure 1:**
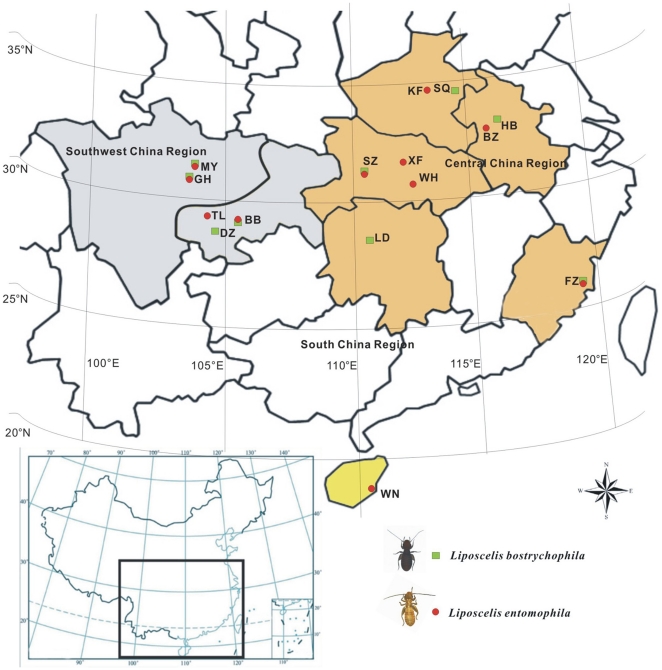
Collection sites of *L. bostrychophila* and *L. entomophila*. Population codes correspond to those in Table 1. The populations of two psocids were divided into three population groups based on Chinese animal fauna. The coloured regions indicate psocid collection provinces. SWCR, coloured gray, includes two provinces; CCR, coloured orange, includes five provinces and SCR, coloured yellow, includes just one province.

Approximately nine populations of *L. bostrychophila* and 11 populations of *L. entomophila* were sampled at grain storage facilities from 15 locations in eight provinces of China from 2008 to 2010, covering three Chinese Animal Fauna Regions [Southwest China Region (SWCR), Central China Region (CCR), and South China Region (SCR)] (Table 1 and 2; Figure 1). All specimens were identified to species initially using morphological characteristics [33] and confirmed by genetic methods using internal transcribed spacer 2 (ITS2) rDNA genes after DNA extraction [10]. The samples were stored at −80°C until DNA extraction. Genomic DNA was extracted from single adult females using the protocols described by Jia et al. (2009) [34] with slight modifications and all DNA samples were stored at −20°C for later use.

**Table 1:** Sampling sites for psocids studied in this study.

Locality (Code)	Latitude	Longitude	Year
Beibei, Chongqing (BB)	29°49′N	106°25′E	2008
Dazu, Chongqing (DZ)	29°42′N	105°43′E	2008
Mianyang, Sichuan (MY)	31°28′N	104°38′E	2008
Guanghan, Sichuan (GH)	30°58′N	104°17′E	2009
Tongliang, Chongqing (TL)	29°50′N	106°3′E	2009
Fuzhou, Fujian (FZ)	26°4′N	119°19′E	2010
Suizhou, Hubei (SZ)	31°40′N	113°21′E	2009
Loudi, Hunan (LD)	27°42′N	111°59′E	2010
Huaibei, Anhui (HB)	33°57′N	116°48′E	2009
Shangqiu, Henan (SQ)	34°25′N	115°39′E	2010
Xiangfan, Hubei (XF)	32°1′N	112°8′E	2009
Wuhan, Hubei (WH)	32°36′N	114°19′E	2008
Kaifeng, Henan (KF)	34°47′N	114°19′E	2008
Bozhou, Anhui (BZ)	34°50′N	115°47′E	2010
Wanning, Hainan (WN)	18°47′N	110°22′E	2010

**Table 2:** Summary statistics observed in *L. bostrychophila* and *L. entomophila* populations in this study.

Species	Region	Code	*Cytb*				ITS			
			*n*	HN	*h*±SD	π±SD	n	HN	*h*±SD	π±SD
*L. bostrychophila*	SWCR	BB	14	4	0.396±0.159	0.00099±0.00044	9	8	0.972±0.064	0.05034±0.00604
		DZ	10	10	1.000±0.045	0.02156±0.00347	8	7	0.964±0.077	0.04004±0.00796
		MY	13	4	0.423±0.164	0.00107±0.00046	12	11	0.985±0.040	0.03469±0.00338
		GH	-	-	-	-	5	4	0.900±0.161	0.02872±0.00701
	CCR	FZ	15	5	0.562±0.143	0.00216±0.00071	-	-	-	-
		SZ	13	2	0.154±0.126	0.00071±0.00058	10	9	0.978±0.054	0.04647±0.00485
		LD	14	7	0.802±0.094	0.02452±0.00407	9	8	0.972±0.064	0.04677±0.00610
		HB	13	8	0.808±0.113	0.00995±0.00599	10	6	0.778±0.137	0.02202±0.00564
		SQ	11	6	0.727±0.144	0.00294±0.00090	11	9	0.964±0.051	0.03727±0.00294
*L. entomophila*	SWCR	BB	15	4	0.371±0.153	0.00123±0.00060	10	8	0.956±0.059	0.02963±0.00446
		TL	16	4	0.442±0.145	0.00244±0.00123	10	8	0.933±0.077	0.02007±0.00282
		GH	14	5	0.505±0.158	0.00277±0.00111	10	6	0.844±0.103	0.01771±0.01003
		MY	16	3	0.492±0.117	0.00179±0.00075	12	10	0.970±0.044	0.01621±0.00351
	CCR	SZ	15	3	0.257±0.142	0.00062±0.00035	11	10	0.982±0.046	0.01809±0.00312
		XF	14	3	0.626±0.104	0.00754±0.00181	10	6	0.778±0.137	0.00811±0.00180
		WH	15	3	0.257±0.142	0.00185±0.00106	15	8	0.733±0.124	0.00469±0.00108
		KF	16	5	0.533±0.142	0.00385±0.00174	11	6	0.727±0.144	0.01421±0.00857
		BZ	-	-	-	-	10	6	0.867±0.085	0.03746±0.00461
		FZ	10	9	0.978±0.054	0.01581±0.00242	12	9	0.909±0.079	0.02902±0.00618
	SCR	WN	17	8	0.728±0.114	0.00268±0.00068	13	11	0.974±0.039	0.02984±0.00575

*n*, number of individuals sequenced; HN, number of different haplotype; *h*, haplotype diversity; *π*, nucleotide diversity.

### Molecular data

The *Cytb* and ITS regions were amplified by PCR. A 485 bp fragment of the mitochondrial *Cytb* gene was amplified using the primer pairs CBF1 (5′-TATGTACTACCATGAGGACAAATATC-3′) and CBR1 (5′- ATTACACCTCCTAATTTATTAGGAAT-3′) [35]; the complete ITS1-5.8 S-ITS2 (ITS) region (∼778 bp) was amplified with 18SF1 (5′-CCGATTGAACGATTTAGTGAGGTCTT-3′) and 28SR1 (5′-TGCTTAAATTCAGCGGGTATTCTCG-3′) [10]. All PCR reactions were carried out in a total volume of 50 µl, utilizing 60 ng of the extracted DNA, 0.4 µM each primer, 100 µM each dNTP, 4 mM Mg^2+^, 10×PCR reaction buffer, ddH_2_O and 2 unit of Taq DNA polymerase (5 U/µl, Takara, China-Japan Joint Company, Dalian, China). PCR reactions were performed on a Bio-RAD S1000™ Thermal Cycler (Bio-Rad Laboratories, Hercules, CA) and consisted of an initial denaturation step at 94°C for 4 min, followed by 35 cycles of 94°C for 30 s, with annealing temperatures of 46°C (*Cytb*) or 60°C (ITS) for 50 s and 72°C for 1 min, with a final 10 min extension at 72°C. PCR products were visualized on 1.0% agarose gels under UV light and purified using a gel band purification kit (Watson, Shanghai, China).

*Cytb* genes were directly sequenced using PCR purified products from both forward and reverse PCR primers. For ITS sequences, the purified products were ligated into pGEM-T Easy vectors (Promega, Madison, WI) followed by ampicillin selection. All the sequences were read on an ABI 3730 automated DNA sequencer (Applied Biosystems, Foster city, CA, USA). Sequences were assembled using Bioedit 7.0.9.0 [36] and manually corrected by eye. The conserved motifs and secondary structure of 5.8 S rRNA genes allowed us to identify putative ITS pseudogenes [12]. The secondary structure of 5.8 S rRNA gene was reconstructed using Mfold Server [37] and helices were numbered in accordance with Wuyts et al. (2001) [38]. The secondary structure and three conserved motifs (M1, M2, and M3) of 5.8 S gene were checked to exclude the putative ITS pseudogenes from our ITS datasets. The unique sequences of both genes were deposited into GenBank under accession numbers JF419805–JF419883 (*Cytb*), and JN828813–JN828948 (ITS).

### Population genetic analyses

The number of haplotypes (*n*), haplotype diversities (*h*), and nucleotide diversities (*π*) for the populations of each species were estimated using the software DnaSP 5.10.00 [39]. Pairwise mismatch distributions and test statistics [the test statistic of raggedness (*rg*) and sum of square deviations (SSD)] were performed using Arlequin 3.1 [40] for all the sampling locations combined to find evidence of past demographic expansions. According to coalescent theory, a population at demographic equilibrium usually exhibits a multimodal mismatch distribution, but is usually unimodal following a recent population demographic or range expansion [41]. A significant SSD value is considered as evidence of departure from the estimated demographic model of a sudden population expansion and small *rg* value represents a population which has experienced sudden expansion [42]. Tajima's *D* [43] and Fu's *F*_S_ [44], were also calculated by Arlequin 3.1 to investigate the historical population demographics and to test whether the sequences conformed to the expectations of neutrality. Expectations of Fu's *F*s, Tajima's *D* are nearly zero in a constant-size population; significantly negative values signify a sudden expansion in population size, whereas significantly positive values indicate processes such as a population subdivision or recent population bottleneck [44].

Pairwise *F*_ST_ and gene flow were calculated using Arlequin 3.1 [40]. Two-level and three-level hierarchical analyses of molecular variance (AMOVA) were conducted to evaluate the possible population genetic structure of *L. bostrychophila* and *L. entomophila* using Arlequin 3.1 with 10,000 permutations. In addition, the correlation between genetic and geographic distances was investigated with a partial Mantel test executed by the web-based program Isolation By Distance Web Service (IBDWS) version 1.52 [45]. The spatial analysis of molecular variance (SAMOVA) was also employed to detect genetic barriers and defines groups of populations that are geographically homogeneous and maximally differentiated from each other, and was performed using SAMOVA 1.0 [46] with 10,000 permutations.

### Phylogenetic analyses and haplotype network construction

Phylogenetic relationships between the *Cytb* haplotypes and ITS haplotypes of the two psocids, *L. bostrychophila* and *L. entomophila*, were performed with a Bayesian phylogenetic inference framework and maximum-likelihood (ML) method, respectively. *L. decolor* (JF440956 and JF742536), was used as an outgroup due to its close relationship with *L. bostrychophila* and *L. entomophila*. First, the coding region *Cytb* sequences were translated into amino acids for confirmation in MEGA 5.01 [47] and examined for the presence of stop codons and other indicators that they were nuclear copies. *Cytb* and ITS haplotypes sequence alignments were conducted with Clustal X Multiple Alignment [48] using the default options. For *Cytb* sequences, transversions and transitions versus sequence divergence were plotted, to evaluate the possibility of sequence saturation in DAMBE 5.2.34 [49]. The best-fit model of nucleotide substitution for mitochondrial and nuclear DNA sequences was determined using the Akaike Information Criterion in jModelTest 0.1.1 [50]. For *Cytb* haplotypes, three partitioned Bayesian analyses were carried out and the best-fit model for 1st, 2nd, and 3rd codon position was HKY+I, TPM3uf+G, HKY+G, respectively. The TPM3uf+I+G substitution model was utilized for the ITS haplotypes data set. Phylogenetic trees for *Cytb* haplotypes data set were reconstructed using MrBayes v3.12 [51]. Four independent Markov chains were simultaneously run for seven million generations (ngen = 7,000,000) with a heating scheme (temp = 0.1). Trees were sampled each 100th generation (samplefreq = 100) and the first 20% of the generations were discarded as burn-in and the remaining samples were used to compute the consensus tree. Stationarity was considered reached when the average standard deviation of split frequencies was below 0.01 [52]. Phylogenetic trees for ITS haplotypes data set were conducted by PhyML 3.0 (http://www.atgc-montpellier.fr/phyml/) [53] using ML algorithms with 100 times bootstrap replications.

Traditional phylogenetic methods assume that ancestral nodes are no longer present in the data set and that the evolution of the data set follows a bifurcating pattern. Intraspecific data sets usually have features, including the persistence of ancestral haplotypes, the existence of multiple descendant haplotypes and often low levels of sequence variation [54], which do not fulfil the assumptions of traditional phylogenetic methods. In the present study, median-joining networks of haplotypes of each of the two genes were constructed using Network 4.5.1.6 [55] to describe relationships among unique haplotypes.

## Results

### Mitochondrial *Cytb* analyses

#### Sequence Variation and Genetic Diversity

A 433 bp fragment of the *Cytb* gene was aligned and analyzed from 251 individuals (including 103 *L. bostrychophila* and 148 *L. entomophila*) and no insertions or deletions were detected in any of the sequences. The aligned sequences of 10 populations of *L. entomophila* contained 55 segregating sites with 60 different polymorphisms representing 28 singleton sites and 27 parsimony informative sites. In comparison, the aligned sequences of eight populations of *L. bostrychophila* had 76 segregating sites with 78 different polymorphisms representing 37 singleton sites and 39 parsimony informative sites.

**Figure 2:**
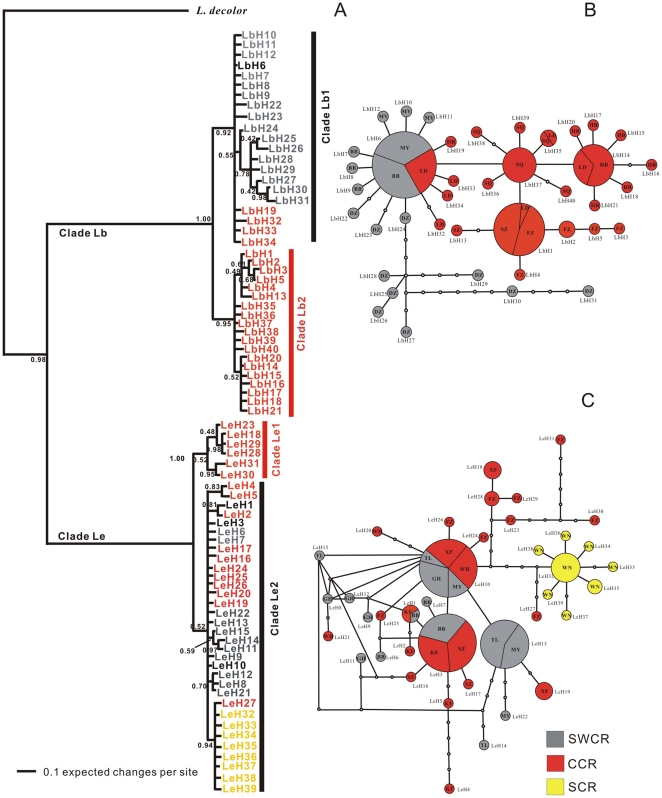
The partitioned Bayesian phylogenetic tree and network of haplotypes for *L. bostrychophila* and *L. entomophila* based on *Cytb* haplotypes. A, the partitioned Bayesian phylogenetic tree for both species; B, Median-joining network of haplotypes for *L. bostrychophila*; C, Median-joining network of haplotypes for *L. entomophila*; Numbers above the branches are Bayesian posterior probabilities (only values above 40% are shown) and taxa are haplotypes. In median-joining network, white dots represent substitutions (lost haplotypes); for each haplotype, the size is proportional to their observed frequencies. The SWCR group is coloured gray, the CCR group is coloured orange and the SCR group is coloured yellow.

In total, 39 haplotypes were detected in *L. entomophila* populations and 40 haplotypes in *L. bostrychophila* populations (Table 2). Among *L. entomophila* haplotypes, LeH1, LeH3, LeH10, and LeH13 were shared by several populations, and the highest haplotype diversity was 0.978 (FZ) and the lowest was 0.257 (SZ, WH) (Table 2). The overall population haplotype diversity for *L. entomophila* was 0.853. Haplotypes LbH1, LbH6, LbH14 and LbH35 were also shared in some of the *L. bostrychophila* populations. The haplotype diversity of all the *L. bostrychophila* populations was 0.875, and the highest was the DZ population (1.000) and the lowest was the SZ population (0.154) (Table 2). The haplotype diversity was not significantly different between these two psocid species (*F*_1, 16_ = 0.598, *P* = 0.451), and the nucleotide diversity of *L. bostrychophila* (*π* = 0.02801±0.00117) was higher than that of *L. entomophila* (*π* = 0.00778±0.00071), but the difference was not significant (*F*_1, 16_ = 1.281, *P* = 0.274). However, the two clades (Clade Lb1 and Clade Lb2) (Figure 2A) were considerably divergent within *L. bostrychophila*, which may affect the results of population genetic diversity estimates. We compared the population genetic diversity of these two psocid species at the same geographic region scale. For the SWCR, the haplotype diversity of *L. bostrychophila* was 0.685, which was not significantly lower than that of *L. entomophila* (*h* = 0.760±0.033) (*F*_1, 5_ = 0.832, *P* = 0.404). However, the nucleotide diversity of *L. bostrychophila* (*π* = 0.00875±0.00341) was not significantly higher than that of *L. entomophila* either (*π* = 0.00360±0.00185) (*F*_1, 5_ = 1.027, *P* = 0.357). For the CCR, both the haplotype and nucleotide diversities of *L. bostrychophila* (*h* = 0.850±0.036; *π* = 0.01151±0.00322) were higher than those of *L. entomophila* (*h* = 0.795±0.035; *π* = 0.00720±0.00111), but not significant (*F*_1, 8_ = 0.196, *P* = 0.670; *F*_1, 8_ = 0.167, *P* = 0.693).

#### Phylogenetic analyses

Substitution saturation of sequences was tested using the software DAMBE 5.0.59. A lack of evidence of saturation means that the diversity was not underestimated and that the sequences were suitable for phylogenetic reconstruction. Therefore, a phylogenetic tree that used three partitioned Bayesian analyses showed that all haplotypes corresponding to *L. bostrychophila* formed to a single well-supported clade designated as clade Lb, while all the haplotypes corresponding to *L. entomophila* formed another single well-supported clade designated as clade Le (Figure 2A). Clade Lb could be subdivided into two well-supported clades (Clade Lb1 and Clade Lb2). Clade Le could also be subdivided into two clades (Clade Le1 and Clade Le2) with low Bayesian posterior probabilities (0.52 and 0.52) (Figure 2A). Clade Lb1 contained five populations and if the haplotypes LbH19, LbH32, LbH33, and LbH34 were excluded, it would correspond with the SWCR (Figure 2A). Clade Lb2 contained five populations which were all included in the CCR. For Clade Le, two clusters were not arranged according to relatively geographic regions. Clade Le1 consisted of six haplotypes (LeH18, LeH23, LeH28, LeH29, LeH30, and LeH31) from two populations, XF and FZ, which were included in the CCR regions. Clade Le2 was admixed in all 10 populations and had no geographical specificity (Figure 2A).

The median-joining network among haplotypes of the *L. bostrychophila* populations, representing genealogical relationships is shown in Figure 2B. The networks of related haplotypes were almost clustered into two areas corresponding to SWCR and CCR. Because LbH6 was shared by three populations belonging to the two regions, the two areas of the network were not distinct. The *L. bostrychophila* haplotype networks were very consistent with the Bayesian phylogenetic tree Clade Lb. For *L. entomophila*, the network suggested little or no association between haplotypes and geography (Figure 2C) which was consistent with the Bayesian phylogenetic tree. The haplotypes of the same population/region did not cluster together. The highest frequency haplotype was LeH10, followed by LeH3 and LeH13, which consisted of 37, 36, and 23 individuals, respectively, and they occupied a central position in the network (Figure 2C).

When the Bayesian tree and median-joining network for haplotypes were combined, the 40 haplotypes of *L. bostrychophila* were divided into two clades that coincided with our predefined regions. The 39 haplotypes of *L. entomophila* were also divided into two clades with low Bayesian posterior probabilities; however, no population-specific clustering pattern was revealed by the Bayesian analysis, but the WN population clustered in the haplotype network.

#### Population structure and Demographic history analyses

AMOVA results showed that there was significant genetic differentiation of *L. bostrychophila* and *L. entomophila* populations at various hierarchical levels (among regions, among populations within regions, and within populations) (Table 3). For *L. bostrychophila*, high genetic structure was found (*Φ*_ST_ = 0.751, *P*<0.001). AMOVA showed that 75.14% of the variation was among populations and only 24.86% was partitioned within populations. When performing a three-level AMOVA to test for structure between SWCR and CCR, we also detected a high genetic structure (*Φ*_CT_ = 0.672, *P*<0.05) between these two groups with a large portion of total genetic structure (67.15% was partitioned among groups). A small portion of the total genetic structure (15.88%) was apportioned among populations and was also significant within a region (*Φ*_SC_ = 0.484, *P*<0.001). For *L. entomophila*, a two-level AMOVA test for structure among populations showed high genetic structure (*Φ*_ST_ = 0.566, *P*<0.001) with slightly more of the total genetic structure apportioned among populations (56.65%). When performing a three-level AMOVA to test for genetic structure among groups, we found that population genetic structure was significant (*Φ*_CT_ = 0.368, *P*<0.05) with a little more portion (36.84%) of total genetic structure. Thus, both of these two species had significant geographic structure, but compared to *L. entomophila*, *L. bostrychophila* had more significant geographic structure. For SAMOVA analysis, the *K* value increased from 2 to 5 for *L. bostrychophila* and *L. entomophila*, respectively. The *F*_CT_ was significant and highest at 5 groups for both species (Table S1). The Mantel test (10,000 randomizations) indicated that there was not a significant IBD effect among *L. bostrychophila* (*r* ^2^ = 0.008, *P* = 0.316) and *L. entomophila* (*r* ^2^ = 0.079, *P* = 0.955) populations.

**Table 3:** Analysis of molecular variance (AMOVA) of *Cytb* and ITS data from the populations of the two psocids species.

Gene	Source of variation	*L. entomophila*				*L. bostrychophila*			
		df	SS	%	Φ Statistic	Df	SS	%	Φ Statistic
*Cytb*	Two level								
	Among populations	9	141.084	56.65		7	461.341	75.14	
	Within populations	138	106.544	43.35	Φ_ST_ = 0.566***	95	157.14	24.86	Φ_ST_ = 0.751***
	Three level								
	Among groups	2	83.611	36.84	Φ_CT_ = 0.368*	1	332.02	67.15	Φ_CT_ = 0.672*
	Among populations within groups	7	57.473	25.23	Φ_SC_ = 0.399*	6	129.321	15.88	Φ_SC_ = 0.484***
	Within populations	138	106.544	37.93	Φ_ST_ = 0.621**	95	157.144	16.96	Φ_ST_ = 0.830***
ITS	Two level								
	Among populations	10	420.982	23.91		7	275.029	11.26	
	Within populations	113	1048.816	76.09	Φ_ST_ = 0.239***	66	1197.079	88.74	Φ_ST_ = 0.113***
	Three level								
	Among groups	2	62.126	−3.92	Φ_CT_ = −0.039	1	31.693	−1.35	Φ_CT_ = −0.013
	Among populations within groups	8	358.856	26.70	Φ_SC_ = 0.257***	6	243.336	12.10	Φ_SC_ = 0.119***
	Within populations	113	1048.816	77.22	Φ_ST_ = 0.228***	66	1197.079	89.25	Φ_ST_ = 0.107***

* *P*<0.05;

** *P*<0.01;

*** *P*<0.001; df, degree of freedom; SS, sum of squares; %, percentage of variation.

For *L. bostrychophila*, pairwise *F*_ST_ ranged from 0.000 to 0.982 and most of the values were statistically significant (Table 4). For *L. entomophila*, pairwise *F*_ST_ ranged from −0.011 to 0.889 and most of the values were statistically significant (Table 5). One *F*_ST_ value was negative, indicating that extremely high gene flow existed between these two populations. Most of the significant and high *F*_ST_ values for both psocids indicate high levels of genetic differentiation among these populations.

**Table 4:** Pairwise *F*_ST_ values (below diagonal) and gene flow (*N*m, above diagonal) among populations for *L. bostrychophila* using *Cytb* sequences.

Population	HB	SZ	SQ	FZ	BB	MY	DZ	LD
HB		0.629	1.777	0.648	0.068	0.070	0.173	0.622
SZ	0.443*		0.371	5.089	0.009	0.009	0.096	0.338
SQ	0.219*	0.574*		0.502	0.021	0.022	0.123	0.440
FZ	0.435*	0.089	0.499*		0.017	0.018	0.096	0.329
BB	0.881*	0.982*	0.960*	0.967*		Inf	0.825	1.302
MY	0.876*	0.981*	0.958*	0.966*	0.000		0.885	1.372
DZ	0.743*	0.839*	0.803*	0.839*	0.377*	0.361*		1.195
LD	0.446*	0.596*	0.532*	0.603*	0.278*	0.267*	0.295*	

* *P*<0.05; Inf = Infinite.

**Table 5:** Pairwise *F*_ST_ values (below diagonal) and gene flow (*N*m, above diagonal) among populations for *L. entomophila* using *Cytb* sequences.

Pop	SZ	XF	WH	KF	TL	GH	BB	MY	WN	FZ
SZ		0.656	0.268	12.818	0.205	0.345	Inf	0.171	0.062	0.569
XF	0.433*		3.280	0.913	1.687	3.274	0.709	1.798	0.215	4.303
WH	0.651*	0.132*		0.600	0.723	30.291	0.333	0.715	0.099	1.067
KF	0.038	0.354*	0.454*		0.401	0.713	95.024	0.391	0.119	0.742
TL	0.709*	0.229*	0.409*	0.555*		0.853	0.243	Inf	0.098	0.885
GH	0.592*	0.132*	0.016	0.412*	0.369*		0.418	0.847	0.117	1.165
BB	0.000	0.414*	0.600*	0.005	0.673*	0.545*		0.214	0.072	0.607
MY	0.745*	0.218*	0.411*	0.561*	–0.011	0.371*	0.701*		0.088	0.874
WN	0.889*	0.699*	0.834*	0.808*	0.836*	0.811*	0.874*	0.851*		0.373
FZ	0.467*	0.104*	0.319*	0.403*	0.361*	0.300*	0.452*	0.364*	0.572*	

* *P*<0.05; Inf = Infinite.

**Figure 3:**
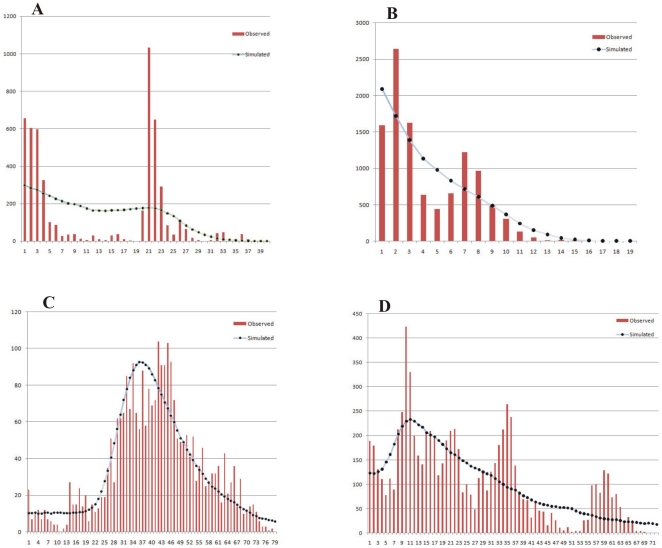
Mismatch distributions of pairwise nucleotide differences for total populations of *L. bostrychophila* and *L. entomophila*. A, B are observed and simulated mismatch distributions for *L. bostrychophila* and *L. entomophila* using the *Cytb* sequences, respectively; C, D are observed and simulated mismatch distributions for *L. bostrychophila* and *L. entomophila* using the ITS sequences, respectively. The horizontal axis represents the number of pairwise differences and the vertical axis represents the relative frequencies of pairwise comparisons.

Demographic history changes were analysed for *L. bostrychophila* and *L. entomophila* populations using two neutrality tests and mismatch distributions. For *L. bostrychophila*, Tajima's *D* and Fu's *F*s values were not significantly negative in all populations pooled in one group or in the CCR group, but were significantly negative in the SWCR group (Table 6). For *L. entomophila*, Tajima's *D* and Fu's *F*s values were significantly negative in all populations pooled in one group, and in the CCR and SCR groups, but not significant in the SWCR group (Table 6). The mismatch distribution did not indicate a rapid demographic expansion, where results were bimodal curves for both of the total *L. bostrychophila* and *L. entomophila* populations (Figure 3A and 3B). For *L. bostrychophila* populations, the test statistics *rg* was significantly low, suggesting that the observed distributions were smoother than the expected under a model of population stability. However, the mismatch population test statistics SSD were small and statistically significant, indicating that the sudden expansion model could not be accepted (Table 7). For *L. entomophila* populations, the mismatch population test statistics *rg* and SSD were small and not statistically significant, indicating that the sudden expansion model could not be rejected (Table 7).

**Table 6:** Results of the neutrality tests calculated for *L. bostrychophila* and *L. entomophila* populations.

Gene	Regions	*L. bostrychophila*		*L. entomophila*	
		Fu's *F_S_*	Tajima's *D*	Fu's *F_S_*	Tajima's *D*
*Cytb*	SWCR	−6.041*	−2.071**	−2.626	−0.458
	CCR	−4.180	−0.903	−8.484**	−1.999**
	SCR	-	-	−4.869***	−2.032**
	Total	−5.979	−0.549	−25.388***	−2.026**
ITS	SWCR	−3.708	−1.099	−2.761	−0.947
	CCR	−2.219	−0.797	−4.134	−0.543
	SCR	-	-	0.582	−0.373
	Total	−11.153*	−1.161	−23.072**	−1.054

* , *P*<0.05;

** , *P*<0.01;

*** , *P*<0.001.

**Table 7:** Values of the mismatch distribution test statistics for *L. bostrychophila* and *L. entomophila* based on *Cytb* and ITS sequences.

Gene	*L. bostrychophila*		*L. entomophila*	
	SSD	*rg*	SSD	*rg*
*Cytb*	0.06123*	0.04541*	0.01869	0.03284
ITS	0.00413	0.00208	0.00257	0.00257

* , *P*<0.05; SSD, sum of squared deviation; *rg*, Harpending's raggedness statistic.

### ITS sequence analyses

**Figure 4:**
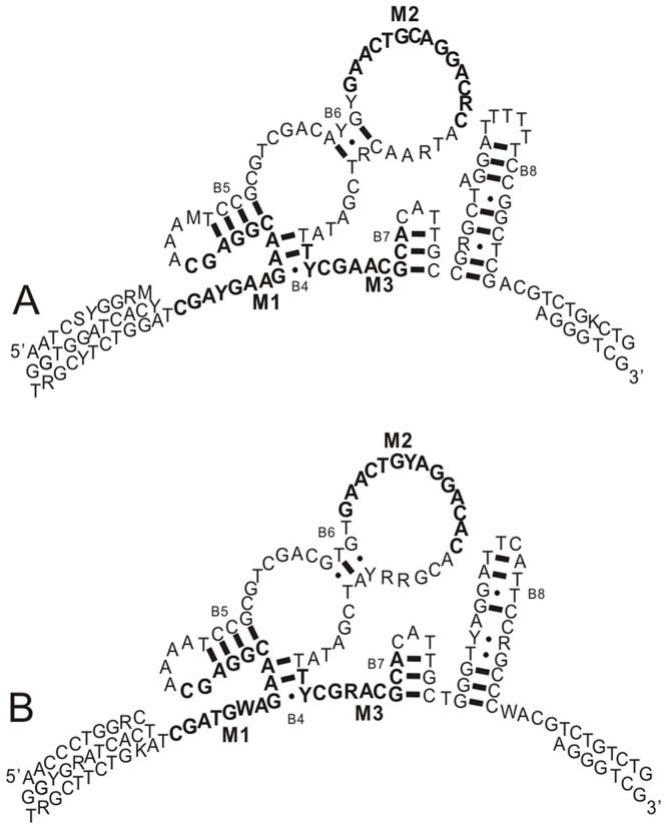
Consensus sequences and secondary structures of the 5.8 S rRNA in *L. bostrychophila* and *L. entomophila*. A: *L. bostrychophila*; B: *L. entomophila*; The helices (B4–B8) are numbered according to Wuyts et al. (2001). Conserved motifs (M1–M3) are in bold black letters.

The complete ITS1-5.8 S-ITS2 sequences varied from 764 to 780 bp and 774 to 782 bp for *L. bostrychophila* and *L. entomophila*, respectively, and were characterized by very high levels of genetic diversity. A fixed length, 154 bp of 5.8 S gene was located in the ITS region for both *Liposcelis* species and the secondary structures of 5.8 S rDNA sequence with three conserved motifs (M1, M2, and M3) were shown in Figure 4.

In total, 74 individuals of *L. bostrychophila* were sequenced for ITS and 59 haplotypes were detected. The difference among the haplotypes was due to 227 polymorphic sites, of which 161 were parsimony informative sites. One hundred and twenty four individuals of *L. entomophila* were also sequenced for ITS and 77 haplotypes were detected and the difference among these haplotypes was due to 155 polymorphic sites, of which 97 were parsimony informative sites. The numbers of haplotype (*n*), diversities of haplotype (*h*) and nucleotide (*π*) for each population of *L. bostrychophila* and *L. entomophila* were summarized in Table 2. The haplotype diversity for all *L. bostrychophila* individuals (0.990) was higher than all of *L. entomophila* individuals (0.975) and the nucleotide diversity of *L. bostrychophila* (*π* = 0.04081±0.00194) was also higher than that of *L. entomophila* (*π* = 0.02569±0.00188).

**Figure 5:**
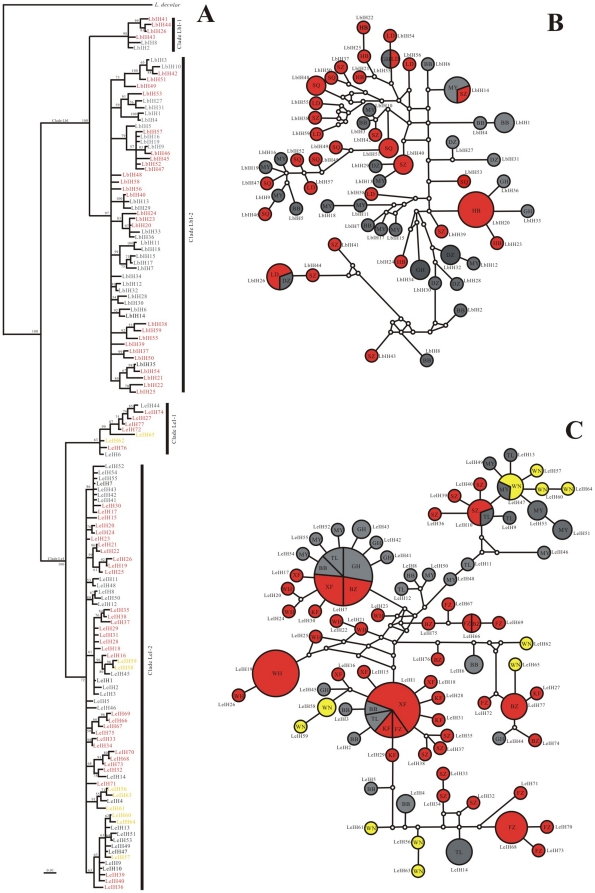
The ML phylogenetic tree and network of haplotypes for *L. bostrychophila* and *L. entomophila* based on ITS haplotypes. A, the ML phylogenetic tree for both species; B, C are median-joining network of haplotypes for *L. bostrychophila* and *L. entomophila*, respectively. Numbers above the branches are bootstrap support values (only values above 50% are shown) and taxa are haplotypes. In median-joining network, white dots represent substitutions (lost haplotypes); for each haplotype, the size is proportional to their observed frequencies. The coloured regions are consistent to Figure 2.

For *L. bostrychophila*, all of the 59 haplotypes were divided into two clusters (CladeLbI-1 and CladeLbI-2) with high support values (98 and 97) and not arranged according to our predefined geographic regions (Figure 5A). For *L. entomophila*, the 77 haplotypes were similar to *L. bostrychophila* and also were not arranged according to geographic regions (Figure 5A). Median-joining networks did not show clear pattern of structure. Haplotypes from the same sampling localities, or predefined geographic regions did not tend to preferentially occupy a particular clade of the network (Figure 5B and 5C). Generally, some haplotypes connected with each other through a few mutations and many missing haplotypes were detected.

Several AMOVAs at different hierarchical levels showed that there were no genetic structures of *L. bostrychophila* and *L. entomophila* populations according to our predefined regions (Table 3). The AMOVA at a three-level analysis showed that most of the variation (89.25% for *L. bostrychophila* and 77.22% for *L. entomophila*) was within populations. For SAMOVA analysis, the *K* value also increased from 2 to 5 for these two species (Table S1). The *F*_CT_ was significantly highest at 2 groups for *L. bostrychophila*. However, the results of SAMOVA analyses indicated that there was lack of genetic structure among *L. entomophila* populations from our samples. In addition, the Mantel test (10,000 randomizations) indicated that there was no significant correlation between the standardized genetic distance [*F*_ST_/(1−*F*_ST_)] and the log of the geographical distance for *L. bostrychophila* (*r* ^2^ = 0.019, *P* = 0.24) and *L. entomophila* (*r* ^2^ = 0.011, *P* = 0.657) populations, indicating that isolation by distance might not be a factor in the gene flow of *L. bostrychophila* and *L. entomophila*. Pairwise estimates of *F*_ST_ among *L. bostrychophila* and *L. entomophila* populations revealed that the genetic differentiation existed between a large portion of *L. bostrychophila* and *L. entomophila* populations (Table S2 and S3).

For all individuals of *L. bostrychophila* and *L. entomophila*, Tajima's *D* values were not significantly negative but Fu's *F*s values were significantly negative (Table 6). The mismatch distribution analysis of overall populations of *L. bostrychophila* and *L. entomophila* showed a multimodal curve (Figure 3C and 3D) and which did not indicate a rapid demographic expansion. The mismatch population test statistics *rg* and SSD for both *L. bostrychophila* and *L. entomophila* populations were small and not statistically significant, as expected with a historically expanding population (Table 7).

## Discussion

In the present study, the significant genetic structure was detected among the examined populations according to our predefined regions by two-level and three-level AMOVA analyses using *Cytb* profiles. However, this result was not indicated by ITS profiles (Table 3). The SAMOVA also shows genetic structure in other population combinations for both psocids using mtDNA marker (Table S1). The lack of genetic structure among *L. entomophila* populations was detected by SAMOVA analyses inferred from ITS dataset. The possible explanation for the homogeneity of populations of *L. entomophila* is the high level of gene flows. Compared to mtDNA makers (lack of recombination), nuclear makers were more sensitive to detect gene flows among the interbreeding populations [56]. The large portions of significant pairwise *F*_ST_ values also provide evidence for population differentiation for both psocids. Intriguingly, the high level of gene flow was also characterized in populations of both species. Although the dispersal ability of psocids seems to be very limited due to their small body size and wingless nature, psocids have great colonization potential and may migrate by human-mediated transport or through air current [32]. This paradoxical phenomenon of significant pairwise *F*_ST_ values and high level of gene flows can be explained by high frequency of inbreeding within a population. The Mantel test revealed a negative non-significant correlation between geographic and genetic distance for both species, which is consistent with previous studies of genetic structure of *L. bostrychophila* [30]. Absence of isolation by distance for both psocids suggests that either the migration is very high that it overcomes the effects of genetic drift, or there has been insufficient time to attain migration-drift equilibrium after recent range expansion [57]. From the above observations, we can conclude that genetic differentiation exists widely in the inter-population for *L. bostrychophila* and *L. entomophila*. It is probable that the genetic differentiation is caused by other factors, such as genetic drift, inbreeding or control practices, and less by geographic distance.

Sequences from mtDNA and nuclear DNA were characterized by very high levels of genetic diversity in asexually and sexually reproducing psocids, *L. bostrychophila* and *L. entomophila*. Especially for *L. bostrychophila*, a high overall genetic variability, as also observed in other populations analysed using either allozymes [19] or RAPDs markers [30]. The haplotype and nucleotide diversity of *L. bostrychophila* was higher than that of *L. entomophila* although the levels of nucleotide diversity were highly variable among different populations for each species. Here, in the comparison of genetic diversities between two psocids, we did not imply the reproduction mode was the key factor in shaping genetic diversity. Actually, high haplotype diversity at a gene locus within populations is determined by many other factors, including large population size, environmental heterogeneity, life-history traits, and origin and ages of the species [58]. Among these factors, environmental heterogeneity is the most key factor [4] and asexual reproduction can also have important consequences for observed levels of sequence polymorphism [59], [60]. Indeed, the higher genetic diversity of mitochondrial genes in asexual populations of psocid, *Echmepteryx hageni* (bark lice) was reported when comparing to its sexual populations [24]. In this study, both psocids were sampled at the same grain facilities (e.g. flour mills or grain storage depots) for each population, and these psocids established their population under the same ecological conditions and pest controlling activities. Both psocids could develop a large population harboured hundreds of thousands of individuals. In this case, although the evolution ages and original place of these two psocids remain unclear, the reproductive systems were worth mentioning. Moreover, nucleotide diversity of *L. bostrychophila* (0.041) and *L. entomophila* (0.026) inferred from ITS sequences were very high compared to other reported ITS sequences nucleotide diversity of invasive insects, such as *Stomoxys calcitrans* and *Cimex lectularius* [42], [56]. On the other hand, populations with very low genetic diversity demonstrated reduced fitness relative to high diversity populations even under permissive conditions [3]. The high genetic diversity of *L. bostrychophila* and *L. entomophila* might explain why these species has a broad tolerance to environmental and habitat stresses and the fast mutational processes inherent in individuals, as well as populations, can enable these two *Liposcelis* species to successfully adapt to complex environments.

Some of the main mechanisms of mutational, ecological and evolutionary change in parthenogenetic insects have been reviewed recently [61], but the mechanism of these high levels of intra-species variability in parthenogenesis is not well understood. It was proposed that larger effective population size, greater mutation rate or possible recent origin of sexual might explain the high genetic diversity of asexual animal populations [24]. Perhaps the most robust explanation for the genetic diversity of *L. bostrychophila* is that this pest is a primarily parthenogenetic psocid occasionally undergoes sexual reproduction, which even at very low frequencies can generate substantial diversity, and that the species is panmictic [62]. Other possibilities, such as numerous random mutations and low genetic drift can also lead to high genetic diversity for *L. bostrychophila* populations (about 17 generations per year).

In addition, many insects are ubiquitously associated with diverse endosymbiotic microorganisms. It is known that some psocids can be infected by endosymbionts [63], [64]. *L. bostrychophila* is parthenogenetic as a result of being infected by a *Wolbachia*-like rickettsial bacterium, which is transovarially transmitted [65]. Many studies suggest that the *Wolbachia* infection has affected the mitochondrial genetic diversity of the host insects and usually reduces the genetic diversity [66], [67]. Therefore, it will be necessary to investigate the effects of *Wolbachia* infection on mtDNA variation in psocids.

For the demographic history analysis, different neutrality tests can be chosen and significantly negative values of neutrality statistics can be indicative of background selection, but are also consistent with either population subdivision or expansion [68]. These processes can be distinguished by comparing different neutrality tests. Fu's *F*s is strongly affected by population expansion or selective sweeps. In various neutrality tests, Fu's *F*s is a particularly powerful test of population growth and it is much more powerful than mismatch distribution in detecting signals of population growth [44]. For all *L. bostrychophila* populations combined, Fu's *F*s and Tajima's *D* values were not significantly negative except significantly negative Fu's *F*s values inferred from ITS, suggesting population expansion in total populations. Due to significantly negative Fu's *F*s and Tajima's *D* values for all *L. entomophila* populations combined (except negative but not significantly Tajima's *D* values inferred from ITS), we speculate that the populations might have undergone population expansion in the past. Evidence from the mismatch distribution statistics and approximate star-like haplotypes median-joining networks also support the hypothesis that *L. bostrychophila* and *L. entomophila* have experienced a historical increase in population size after a period of having a small effective population. The pattern of genetic variability with high haplotype diversity but relatively low nucleotide diversity in *Cytb* gene also suggests the population has experienced population expansion [57]. The test statistics *rg* and SSD failed to reject the null hypothesis of population expansion (Table 7) although the distributions appeared to be multimodal (Figure 3). The multimodal mismatch distributions might due to small sample sizes. The small sample sizes was more confirmed by haplotypes networks which shown many haplotypes were lost (Figure 2B and C, and Figure 5B and C).

A thorough understanding of *L. bostrychophila* and *L. entomophil*a population genetics, including gene flow patterns, geographical origin (source populations), dispersal of source populations and the resultant genetic structure among populations within China is useful for proposing successful integrated pest management tactics for these pests of grain storage systems. However, we realized that the small sized samples used in this study and populations examined here do not represent the true distribution of these two psocids in China. Also, only mtDNA gene and ITS gene were used as the molecular marker and their short length contains limited information for population genetic analyses. Nuclear genes, such as microsatellite DNA need to be examined to gain a deeper understanding of the population genetics and invasion biology of these two psocids. Indeed, microsatellite loci have already been isolated for *L. bostrychophila* and *L. entomophila* [31] and also a large number of microsatellites have been isolated for these two species in our laboratory [69] and these microsatellite loci will be used to further study psocid intra- and inter-specific differentiation and gene flow by sampling more populations and individuals across their distribution range in China.

## Supporting Information

Table S1 **Fixation indices corresponding to groups of populations inferred by SAMOVA analysis for two species.**
(DOC)

Table S2 **Pairwise** ***F*** **_ST_ values (below diagonal) and gene flow (** ***N*** **m, above diagonal) among populations for** ***L. bostrychophila*** **using ITS sequences.**
(DOC)

Table S3 **Pairwise** ***F*** **_ST_ values (below diagonal) and gene flow (** ***N*** **m, above diagonal) among populations for** ***L. entomophila*** **using ITS sequences.**
(DOC)
