# Supplementary material for: Population Genetics of Two Asexually and Sexually Reproducing Psocids Species Inferred by the Analysis of Mitochondrial and Nuclear DNA Sequences
Source: PLoS One. 2012 Mar 27;7(3):e33883. doi: 10.1371/journal.pone.0033883 (PMC3313955; doi:10.1371/journal.pone.0033883)
Supplement: Table S1 — Fixation indices corresponding to groups of populations inferred by SAMOVA analysis for two species. (DOC) [file pone.0033883.s001.doc]

**Table S1 Fixation indices corresponding to groups of populations inferred by SAMOVA analysis for two species**

| Gene | Species | K | Group composition | *F*_CT_ | *F*_SC_ | *F*_ST_ |
| --- | --- | --- | --- | --- | --- | --- |
| *Cytb* | *L. entomophila* | 2 | (WN)( FZ,KF,BB,SZ,TL,MY,GH,WH,XF) | 0.631 | 0.398** | 0.778** |
|  |  | 3 | (WN)(FZ)(others) | 0.586* | 0.352** | 0.732** |
|  |  | 4 | (WN)(FZ)(KF,BB,SZ)(others) | 0.581** | 0.145** | 0.642** |
|  |  | 5 | (WN)(FZ)(TL,MY)(KF,BB,SZ)( others) | 0.591** | 0.040** | 0.608** |
|  | *L. bostrychophila* | 2 | (LD,BB,MY,DZ)(FZ, SZ, HB, SQ) | 0.749* | 0.328** | 0.831** |
|  |  | 3 | (DZ)(BB,MY,LD)(others) | 0.748* | 0.265** | 0.814** |
|  |  | 4 | (DZ )(LD)(BB,MY)(others) | 0.758** | 0.172** | 0.799** |
|  |  | 5 | (DZ)(LD)(BB,MY)(HB,SQ)(others) | 0.764* | 0.036** | 0.773** |
|  |  |  |  |  |  |  |
| ITS | *L. entomophila* | 2 | (KF,FZ,SZ,WN)(BB,TL,XF,GH,WH,MY,BZ) | 0.054 | 0.208** | 0.251** |
|  |  | 3 | (GH,MY)(BB,TL,XF,SZ)(others) | -0.023 | 0.244** | 0.227** |
|  |  | 4 | (WN)(GH,MY,XF)(BB,KF,SZ)(others) | 0.013 | 0.224** | 0.234** |
|  |  | 5 | (WN,WH)(GH,MY)(KF,FZ)(SZ)(others) | -0.05 | 0.266** | 0.227** |
|  | *L. bostrychophila* | 2 | (HB,GH)(others) | 0.142* | 0.058** | 0.192** |
|  |  | 3 | (HB)(GH)(others) | 0.109* | 0.070** | 0.171** |
|  |  | 4 | (HB)(GH)(MY,SQ)(others) | 0.109* | 0.070** | 0.171** |
|  |  | 5 | (HB)(GH)(MY,SQ)(BB)(others) | 0.096* | 0.043** | 0.135** |

* *P* < 0.05; ** *P* < 0.001; K, number of group; *F*_SC_, proportion of total genetic variance due to differences between populations within each group; *F*_ST_, proportion of total genetic variance due to differences between populations; *F*_CT_, proportion of total genetic variance due to the differences between groups. The abbreviations of different populations are explained in Table 1.
