# Supplementary material for: Population Genetics of Two Asexually and Sexually Reproducing Psocids Species Inferred by the Analysis of Mitochondrial and Nuclear DNA Sequences
Source: PLoS One. 2012 Mar 27;7(3):e33883. doi: 10.1371/journal.pone.0033883 (PMC3313955; doi:10.1371/journal.pone.0033883)
Supplement: Table S2 — Pairwise F ST values (below diagonal) and gene flow ( N m, above diagonal) among populations for L. bostrychophila using ITS sequences. (DOC) [file pone.0033883.s002.doc]

**Table S2 Pairwise *F*_ST_ values (below diagonal) and gene flow (*N*m, above diagonal) among populations for *L.* *bostrychophila* using ITS sequences**

| Population | GH | BB | MY | DZ | SQ | SZ | HB | LD |
| --- | --- | --- | --- | --- | --- | --- | --- | --- |
| GH |  | 1.387 | 1.809 | 2.090 | 0.829 | 1.093 | 5.641 | 1.326 |
| BB | 0.153^*^ |  | 3.683 | 6.038 | 2.895 | 6.848 | 0.789 | 4.494 |
| MY | 0.121^*^ | 0.064 |  | 5.618 | 10.059 | 2.705 | 0.977 | 2.419 |
| DZ | 0.106^*^ | 0.039 | 0.043 |  | 2.503 | 11.132 | 1.075 | 5.017 |
| SQ | 0.232^*^ | 0.079 | 0.024 | 0.091^*^ |  | 2.888 | 0.593 | 3.166 |
| SZ | 0.186^*^ | 0.035 | 0.085^*^ | 0.022 | 0.079 |  | 0.707 | Inf |
| HB | 0.042 | 0.240^*^ | 0.204^*^ | 0.189^*^ | 0.297^*^ | 0.261^*^ |  | 0.796 |
| LD | 0.159^*^ | 0.053 | 0.094^*^ | 0.047 | 0.073 | -0.031 | 0.239^*^ |  |

**P* < 0.05; Inf = Infinite.
