# Supplementary material for: Population Genetics of Two Asexually and Sexually Reproducing Psocids Species Inferred by the Analysis of Mitochondrial and Nuclear DNA Sequences
Source: PLoS One. 2012 Mar 27;7(3):e33883. doi: 10.1371/journal.pone.0033883 (PMC3313955; doi:10.1371/journal.pone.0033883)
Supplement: Table S3 — Pairwise F ST values (below diagonal) and gene flow ( N m, above diagonal) among populations for L. entomophila using ITS sequences. (DOC) [file pone.0033883.s003.doc]

**Table S3 Pairwise *F*_ST_ values (below diagonal) and gene flow (*N*m, above diagonal) among populations for *L. entomophila* using ITS sequences**

| Population | TL | GH | BB | MY | XF | KF | FZ | BZ | SZ | WH | WN |
| --- | --- | --- | --- | --- | --- | --- | --- | --- | --- | --- | --- |
| TL |  | 0.756 | 5.392 | 2.558 | 0.576 | 1.085 | 1.882 | 0.729 | Inf | 0.324 | 19.352 |
| GH | 0.249^*^ |  | 1.375 | 0.732 | Inf | 1.021 | 0.539 | 1.157 | 0.503 | 0.486 | 0.724 |
| BB | 0.044 | 0.154^*^ |  | 1.049 | 1.017 | 3.905 | 2.119 | 1.629 | 2.748 | 0.511 | 18.703 |
| MY | 0.089 | 0.255^*^ | 0.192^*^ |  | 0.525 | 0.659 | 0.498 | 0.564 | 1.784 | 0.281 | 2.109 |
| XF | 0.303^*^ | -0.003 | 0.197^*^ | 0.322^*^ |  | 1.127 | 0.402 | 0.509 | 0.422 | 0.347 | 0.590 |
| KF | 0.187^*^ | 0.197^*^ | 0.060 | 0.275^*^ | 0.181^*^ |  | 0.664 | 0.722 | 0.953 | 0.404 | 1.374 |
| FZ | 0.117^*^ | 0.317^*^ | 0.106^*^ | 0.334^*^ | 0.384^*^ | 0.274^*^ |  | 0.836 | 1.083 | 0.275 | 1.561 |
| BZ | 0.255^*^ | 0.178 | 0.133 | 0.307^*^ | 0.329^*^ | 0.257^*^ | 0.230^*^ |  | 0.553 | 0.302 | 1.055 |
| SZ | -0.017 | 0.332^*^ | 0.083 | 0.123^*^ | 0.372^*^ | 0.208^*^ | 0.189^*^ | 0.311^*^ |  | 0.271 | 15.842 |
| WH | 0.436^*^ | 0.339^*^ | 0.328^*^ | 0.470^*^ | 0.419^*^ | 0.383^*^ | 0.476^*^ | 0.453^*^ | 0.480^*^ |  | 0.396 |
| WN | 0.013 | 0.257^*^ | 0.013 | 0.106^*^ | 0.298^*^ | 0.154^*^ | 0.139^*^ | 0.192^*^ | 0.016 | 0.387^*^ |  |

**P* < 0.05; Inf = Infinite.
